# Living in urban or rural environments affect the sleep quality of the elderly in Bushehr (Southern Iran): emphasizing the active and inactive of the elderly

**DOI:** 10.1186/s12889-024-18747-9

**Published:** 2024-05-18

**Authors:** Ahmad Delbari, Fatemeh Ahmadi, Abdossaleh Zar, Atousa Zandvakili, Hamid Reza Sadeghipour, Jamie Sims

**Affiliations:** 1 Research Center on Aging, University of social Welfare and Rehabilitation Sciense, Tehran, Iran; 2 Research Center of Persian Gulf Sports, Nutrition and Health, School of Literature and Humanities, Persian Gulf University, Boushehr, Iran; 3 Department of Sport Science, School of Literature and Humanities, Persian Gulf University, Boushehr, Iran; 4 Department of Sport, Health Sciences, and Social Work, Faculty of Health and Life Sciences, Oxford Brookes University, Oxford, OX3 0BP UK

**Keywords:** Urban environment, Rural environment, Sleep quality, Elderly

## Abstract

**Introduction:**

Sleep disorders have a significant negative impact on mental and physical health, especially among the elderly. Various factors can affect the sleep quality of elderly people. The aim of this research to investigate the effect of urban and rural environments on the sleep quality of elderly people with emphasis on physical activity.

**Method:**

Four hundred and thirty-nine elderly people (226 city residents and 213 village residents) in urban and rural areas of Bushehr (Southern Iran), volunteered to participate in the present study. Information was collected via the General information questionnaire and Petersburg Sleep Questionnaire.

**Result:**

The results showed that active elderly women (*p* < 0.001), and total active elderly (male + female) (*p* < 0.001) living in urban areas compared to inactive elderly and also in rural areas active elderly women (*p* < 0.001), active elderly men (*p* < 0.001) and total active elderly (male + female) (*p* < 0.001) had better overall sleep quality in compared to inactive elderly. Also, elderly men (*p* < 0.001) and the total elderly (male + female) (*p* < 0.001) living in urban areas had better sleep quality than the elderly in rural areas.

**Conclusion:**

Based on the findings, it can be concluded that the way of life (being active) as well as the living environment can affect the sleep quality of elderly people, so that active elderly people and also elderly people living in urban environments had better sleep quality.

## Highlights

• Active elderly people have better sleep quality than inactive elderly people.

• In both urban and rural areas, people who are more physically active have better sleep quality.

• Improving the level of physical activity can be one of the best options to increase the quality of sleep and the health of elderly.

## Introduction

Sleep is a suitable process for recovery, renewal, and restoration of the function of the body’s nervous and physiological system. In fact, sleep is a physiological behavior and a part of every person’s daily life [1]. Research has shown that sleep disorders are the third most common problem in the elderly after headaches and digestive problems. In this regard, behavioral disorders during sleep such as frequent awakenings during night sleep, early awakening, snoring and reduced sleep hours have been reported in the elderly [2]. According to the World Health Organization (WHO), between 2015 and 2050, the proportion of the world’s population over 60 years of age will almost double, from 12 to 22% [3]. In Iran, the percentage of the elderly population has increased from 7.27 to 8.65% between 2006 and 2016, and it is predicted to reach 21.7% in 2050 [4].

Sleep disorders have a significant negative impact on mental and physical health, especially among the elderly [5]. It has been reported that this disorder can cause mental problems such as impaired concentration, irritability, anxiety, and memory impairment, as well as physical problems such as fatigue, blood pressure, obesity, and decreased performance [6]. Sleep disorders are diverse in origin and usually result in excessive daytime sleepiness, which can greatly affect a person’s daytime performance and safety. Also, chronic sleep disorder is associated with increased morbidity and mortality [7]. Proper physical activity and proper cultivation of sleeping and waking patterns are among the features that are effective in the field of success and reducing problems in old age [8]. There is a relationship between sporting activity and sleep, and sleep can have positive effects on health, as studies have shown that people who have sleep-related problems and who are physically inactive can increase their quality of sleep by participating in sports activities [9]. Exercise and physical activity have an effective effect on strength, muscular endurance, cardiovascular endurance, flexibility, speed, agility and balance and improve the quality of life of the elderly [10].

Scientific studies show that the low quality of sleep causes an increase in the strength and speed of heart contraction and an increase in the need for oxygen, as well as the secretion of epinephrine and norepinephrine and stimulation of the sympathetic system, resulting in an increase in the number of breaths, the occurrence of arrhythmia, exacerbation of ischemia and infarction, and in Finally, it becomes a heart attack [11]. In this regard, the results of a research study showed that daily walking has a positive effect on the quality and time of sleep [12]. Also, in a systematic review study that was conducted on elderly people over 60 years of age, it was shown that carrying out exercise programs has a positive effect on various aspects of sleep in the elderly [13]. Riemann et al. (2015) pointed out that several factors such as gender, age, employment status, marital status, socioeconomic class, place of residence, physical and mental health, and nutritional status can be effective in causing sleep disorders. They stated that among the mentioned factors, age has the biggest contribution in causing sleep problems, which due to the increase in the elderly population, sleep problems become more apparent day by day [14]. An active lifestyle is directly related to the physical and mental health of people. Many diseases such as obesity, heart diseases, diabetes, back pain, depression, etc. are caused by an inactive lifestyle. For this reason, having the minimum level of physical activities that can have a positive effect on the health level of people puts people in the spectrum of active lifestyle. However, one of the topics that has a great impact on the quality of life of the elderly is their level of physical fitness and their level of physical activity reflects the lifestyle of the elderly. Research has shown that physical activity can significantly affect the components affecting the health of the elderly, so that in previous research, people who had a higher level of physical activity had a higher quality of closeness [15].

Several studies have also shown that regular physical activity improves sleep patterns and affects the quality of life of the elderly. It has also been shown that physical activity has a positive effect on increasing the functional levels of some lung volumes and capacities, and as a result, improving the quality of sleep [16, 17]. In contrast, Banno et al. (2018) showed in their review study that exercise and physical activity with different intensities in different age groups do not have a significant effect on sleep quality [18]. In a review study by Kovacevic et al. (2017), it was also reported that only resistance exercises had the greatest effect on sleep quality, and when these exercises were combined with aerobic exercises, their effects decreased [19].

However, the quality of sleep directly affects the quality of life and it seems that this issue is multi-dimensional and related to different mental and objective aspects. Research findings have shown that there is a direct relationship between the living environment and the factors affecting the quality of sleep and quality of life, because these environmental factors have a direct effect on the health of people by affecting the state of culture and economy, for this reason the living environment (urban or rural) can indirectly affect people’s sleep quality [20]. Factors such as the impossibility of sports activities for both groups of men and women in public environments are also due to cultural attitudes, which may have an impact on the difference in the level of living and, as a result, the quality of their sleep.

In the review article of Mortazavi et al. (2021), environmental factors such as economic level, staying in a nursing home, living in an environment with extreme cold or heat, unsuitable physical environments and living in certain areas were reported as effective factors in the sleep disorder of the elderly [21]. Ashghab et al. (2022) reported that manipulating environmental factors such as reducing the amount of noise, dimming the ambient light, and using blindfolds and earmuffs can significantly improve sleep quality in nurses [22]. Sleep problems are common worldwide and are more common in urban dwellers and the elderly. Also, it has been reported that sleep disorders and the use of sleeping pills are more common among women and increase with age [23]. In this regard, the results of Mandal et al.‘s study (2018) showed that the prevalence of poor-quality sleep is higher in the urban population than in the rural population. In addition, adult women were reported to suffer from poor quality sleep more than adult men in both urban and rural areas [24].

Investigating physical activity and its effect on sleep quality in the elderly population of urban and rural areas is considered a valuable reference point for other related researches in the society. Chang et al. (2020) reported that less satisfaction with public spaces in the residents of the urban environment through the impact on stress negatively affected the quality of life in both physical and psychological aspects, while the low quality of the living environment through increased stress and poor sleep on everyone Quality of life domains were negatively affected [25]. Also, it causes policy making and planning in the field of improving health in this group of people. It seems that the lifestyle in urban and rural areas can affect physical activity and as a result the quality of sleep. Considering the vast geographical area of Iran and the lack of access of researchers to the same topic by examining the quality of sleep of urban and rural of women and men in the south of Iran. Therefore, the purpose of this study is to investigate the quality of sleep of elderly men and women in rural and urban areas, as well as to investigate the effect of physical activity on the quality of sleep of the elderly in urban and rural areas. Based on this, the main questions of the research are: Can the living environment (rural and urban) affect the quality of sleep of elderly men and women? Can the activeness of elderly men and women affect the quality of sleep of the elderly in urban and rural environments?

## Methods

### Study design

**Fig. 1:**
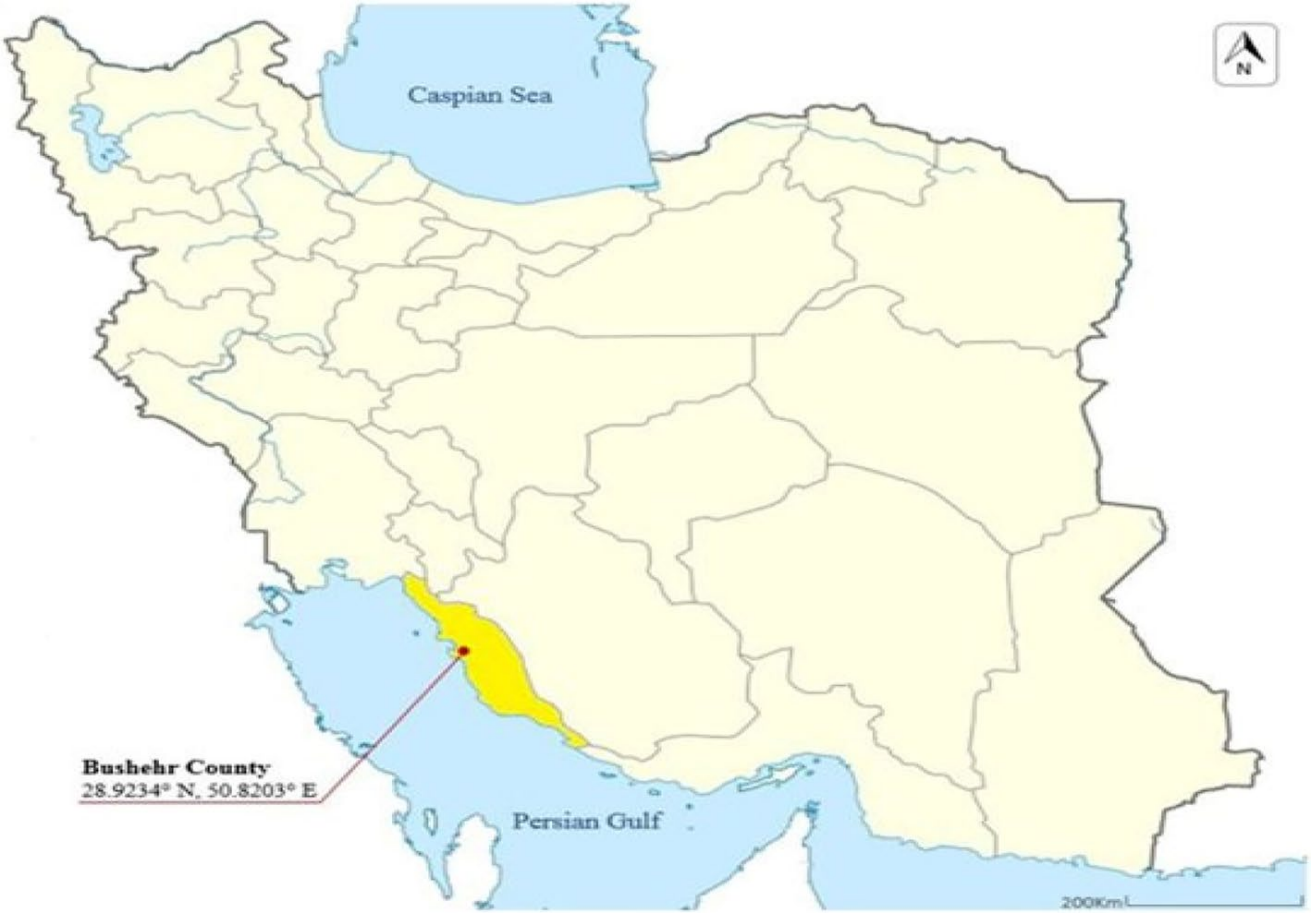
The geographical area was determined in the study (Map of Iran, Bushehr county location in Bushehr province)

The present study is an objective, applied type, and the data was collected using a descriptive-survey method [26]. This project was conducted on Iranian elderly living in both Urban and rural areas of Bushehr in 2023. Four hundred and thirty-nine elderly people from Bushehr voluntarily participated in this study (Fig. 1) (at distance of 1046 km South of Tehran, Iran).

The research samples were divided into active and inactive groups based on self-reported physical activity, so that individuals who participated in at least 3 sessions per week (45 min per session) of physical activity were included in the “physically active” group and people who did not engage in any physical activity (other than daily activities) were included in the inactive group [27]. Accordingly, People who had one or two sessions of physical activity per week were excluded from the study and only people who did not have any physical activity (inactive group) or people who had at least 3 sessions of physical activity (active group) selected and entered the study. First, the demographic characteristics of the subjects such as age, height and weight were recorded. Data were then collected using questionnaires: The Petersburg Sleep Questionnaire and the Demographic Characteristics Questionnaire. The criteria for inclusion of study were being over 60 years old and willing to participate voluntarily. Also, in order to consider ethical considerations, all questionnaires and results reports were without mentioning the names of the research participants.

### Data collection methodology

In order to collect information, the following questionnaires will be used:

#### General information questionnaire

General information checklist includes general questions (age, gender, place of residence).

#### Petersburg sleep questionnaire

To measure sleep quality, the Petersburg Sleep Questionnaire was used which included 9 questions that are categorized into 7 clinically derived components of (1) sleep duration, (2) sleep disturbance, (3) sleep latency, (4) daytime dysfunction due to sleepiness, (5) sleep efficiency, (6) overall sleep quality, and (7) sleep medication use [28]. It examines in the morning. All the above components are calculated based on the results of the questionnaire. The score of each question is in the form of a Likert-type and between 0 and 3, and a score of 3 on each scale indicates the maximum negative. The overall score of this questionnaire is between 0 and 21, and high scores indicate low sleep quality. A score above 5 indicates poor sleep quality. Its reliability and validity have been confirmed in various studies, which have a reliability of 0.83 and validity between 86.5 and 89.6 [29].

### Participant demographics

The age, height and weight of the subjects living in the urban environment (*n* = 226) were respectively equal to equal to 68.61 ± 1.99 Year, 169.14 ± 3.54 centimeter and 67.58 ± 2.17 kg. Also, the age, height and weight of the subjects living in the rural environment (*n* = 213) were respectively equal to equal to 68.86 ± 1.69 Year, 169.07 ± 2.63 centimeter and 67.58 ± 1.97 kg.

### Ethical considerations

All subjects completed their informed consent and were assured that their information would remain confidential. This study was performed in accordance with the principles described in the Declaration of Helsinki (1986) and Approval was taken from the Ethics Committee of Aging Research Center of University of social Welfare and Rehabilitation Science (IR.USWR.REC.1401.100).

### Data analysis procedures

The Kalmogorov-Smirnov test was used to evaluate the normality of the distribution of findings. Also, Independent t-test was used to compare the mean variables obtained from two different groups. statistical analyses were performed using IBM SPSS Statistics 18.0 software (Armonk, NY: IBM Corp.) with a significance level of α = 0.05 which was considered significantly different.

## Results

### Comparison of sleep quality of active and inactive elderly living in urban areas

The results of independent t test (Table 1) showed that urban active elderly men scored lower than inactive urban elderly men in the sleep efficiency (*p* < 0.001), sleep disturbances (*p* = 0.019) and as a result, they are in a better condition. But in other scales, there is no significant difference between two groups of active and inactive urban elderly men.

It was found that active urban elderly women in the subjective quality of sleep scales (*p* < 0.001), sleep latency (*p* < 0.001), sleep efficiency (*p* < 0.001), sleep disturbance (*p* < 0.001), the sleep medication use (*p* < 0.001) and the daytime dysfunction (*p* < 0.001) scored lower than inactive elderly women. Inactive urban people have obtained a lower score and as a result have a better situation. In general, it was found that there is a significant difference between the total sleep quality score of active urban elderly women and inactive urban women, and active urban elderly women have better sleep quality than inactive urban women (*p* < 0.001) (Table 1).

The findings showed that urban active elderly (male + female) in the scales of subjective quality of sleep (*p* = 0.008), sleep latency (*p* < 0.001), sleep efficiency (*p* < 0.001), sleep medication use (*p* < 0.001), daytime dysfunction due to sleepiness (*p* < 0.001), have scored lower than the inactive urban elderly (female + male) and as a result have a better condition. In general, it was found that there is a significant difference between the overall sleep quality score of active urban elderly (female + male) and inactive urban elderly (female + male) and active urban elderly (female + male) have quality. They sleep better than the elderly (male + female) who are inactive in the city (*p* < 0.001) (Table 1).

**Table 1:** Comparison of sleep quality of active and inactive elderly living in urban areas^a^

Variable	Subject	Active	Inactive	*p* value
Mental Quality Of Sleep	Men	0.727±0.69	0.580±0.574	0.213
	Women	1.266±0.643	1.802±0.838	< 0.001*
	Total	1.014±0.719	1.297±0.954	0.008*
Sleep Latency	Men	0.731±0.863	0.803±0.916	0.663
	Women	0.421±0.497	1.831±0.827	< 0.001*
	Total	0.566±0.707	1.401±1.001	< 0.001*
Sleep Duration	Men	1.209±0.729	1.058±0.881	0.326
	Women	1.013±0.528	1.140±0.388	0.096
	Total	1.104±0.635	1.106±0.640	0.983
Sleep Efficiency	Men	0.343±0.565	0.921±0.976	< 0.001*
	Women	1.118±0.631	1.816±0.945	< 0.001*
	Total	0.755±0.714	1.442±1.052	< 0.001*
Sleep Disturbance	Men	0.761±0.552	0.529±0.504	0.019*
	Women	0.868±0.340	1.140±0.350	< 0.001*
	Total	0.818±0.454	0.885±0.517	0.267
Sleep Medication Use	Men	1.253±0.765	1.235±0.586	0.882
	Women	0.802±0.611	1.380±0.724	< 0.001*
	Total	1.014±0.721	1.319±0.671	< 0.001*
Daytime Dysfunction Due To Sleepiness	Men	0.686±0.820	0.686±0.677	0.998
	Women	0.513±0.503	1.718±0.453	< 0.001*
	Total	0.594±0.673	1.286±0.754	< 0.001*
Overall Sleep Quality	Men	5.701±1.605	5.823±1.946	0.717
	Women	6.026±1.540	10.845±2.011	< 0.001*
	Total	5.874±1.573	8.745±3.176	< 0.001*

Data are presented as the mean ± standard error of the mean

*P-value ≤ 0.05 considered significant

^a^Independent t-test was used

### Comparison of sleep quality of active and inactive elderly living in rural areas

The results of independent t test (Table 2) showed that active rural elderly men in the scales of mental quality of sleep (*p* < 0.001), sleep latency (*p* = 0.001), disappearance of sleep (*p* < 0.001), sleep medication use (*p* < 0.001) and daytime dysfunction due to sleepiness (*p* < 0.001) compared to inactive rural elderly men, have scored lower and as a result, they are in a better condition. In general, it was found that there is a significant difference between the overall sleep quality score of active rural men and inactive rural men, and active rural men have better sleep quality than inactive rural men (*p* < 0.001). But in other scales, there is no significant difference between two groups of active and inactive rural elderly men (Table 2).

It was found that active rural elderly women in subjective sleep quality scales (*p* < 0.001), sleep latency (*p* < 0.001), sleep disorders (*p* = 0.003), and daytime dysfunction due to sleepiness (*p* < 0.001) compared to inactive rural elderly women, they have obtained a lower score and as a result, they are in a better condition. In general, it was found that there is a significant difference between the total sleep quality score of active rural elderly women and inactive rural women, and active rural elderly women have better sleep quality than inactive rural women (*p* < 0.001) (Table 2).

The findings showed that active rural elderly (male + female) in the scales of mental quality of sleep (*p* < 0.001), sleep latency (*p* < 0.001), sleep efficiency (*p* = 0.043), sleep disorders (*p* < 0.001), sleep medication use (*p* < 0.001) and daytime dysfunction due to sleepiness (*p* < 0.001) compared to inactive elderly (male + female) Villagers have obtained a lower score and as a result have a better situation. In general, it was found that there is a significant difference between the overall sleep quality score of active rural elderly (female + male) and inactive rural elderly (female + male) and active rural elderly (female + male) have quality They sleep better than the elderly (male + female) who are inactive in rural areas (*p* < 0.001) (Table 2).

**Table 2:** Comparison of sleep quality of active and inactive elderly living in rural areas^a^

Variable	Subject	Active	Inactive	*p* value
Mental Quality Of Sleep	Men	1.326±0.473	1.920±0.633	< 0.001*
	Women	0.852±0.357	1.872±0.668	< 0.001*
	Total	1.070±0.476	1.895±0.649	< 0.001*
Sleep Latency	Men	0.203±0.450	2.300±0.735	0.001*
	Women	0.852±0.872	2.160±0.986	< 0.001*
	Total	0.547±0.775	2.226±0.875	< 0.001*
Sleep Duration	Men	1.203±0.736	1.140±0.452	0.593
	Women	1.327±0.597	1.232±0.466	0.334
	Total	1.269±0.666	1.188±0.460	0.292
Sleep Efficiency	Men	1.185±0.646	1.420±1.051	0.178
	Women	0.918±0.881	1.196±1.068	0.129
	Total	1.043±0.787	1.301±1.061	0.043*
Sleep Disturbance	Men	0.537±0.539	1.140±0.452	< 0.001*
	Women	0.836±0.610	1.125±0.384	0.003*
	Total	0.695±0.594	1.132±0.415	< 0.001*
Sleep Medication Use	Men	0.611±0.492	1.360±0.562	< 0.001*
	Women	1.327±0.597	1.267±0.485	0.551
	Total	0.991±0.655	1.311±0.523	< 0.001*
Daytime Dysfunction Due To Sleepiness	Men	0.203±0.450	1.960±0.637	< 0.001*
	Women	0.852±0.703	1.785±0.802	< 0.001*
	Total	0.547±0.678	1.867±0.731	< 0.001*
Overall Sleep Quality	Men	5.259±1.305	11.240±1.684	< 0.001*
	Women	6.967±2.175	10.642±1.803	< 0.001*
	Total	6.165±2.004	10.924±1.765	< 0.001*

Data are presented as the mean ± standard error of the mean

*P-value ≤ 0.05 considered significant

^a^Independent t-test was used

### Comparing the sleep quality of the elderly in urban and rural areas

In Table 3, the difference in physical activity levels (active and inactive) is not discussed. Rather, the purpose of this table is only to present gender differences between rural and urban areas.

The results of independent t test (Table 3) showed that elderly men in urban areas in the scales of mental quality of sleep (*p* < 0.001), sleep latency (*p* = 0.002), sleep efficiency(*p* < 0.001), Sleep Disturbance (*p* = 0.030), sleep medication use (*p* = 0.002) and daytime dysfunction due to sleepiness (*p* = 0.004) scored lower than elderly men in rural areas, and as a result, they are in a better condition. It was found that there is a significant difference in overall sleep quality score between elderly men on the urban areas and rural areas (*p* < 0.001). The elderly men on the urban areas in the scales of overall sleep quality scored lower than elderly men in rural areas, and as a result, they are in a better condition (Table 3).

Also, it was found that elderly women in urban areas in the scales of mental quality of sleep (*p* = 0.044), sleep latency (*p* = 0.005), Sleep Duration (*p* = 0.001), sleep efficiency (*p* < 0.001) and sleep medication use (*p* = 0.006) scored lower than elderly women in rural areas, and as a result, they are in a better condition. And also, it was found that there is a not significant difference in the overall sleep quality score between women in rural areas and urban areas (*p* = 0.292) (Table 3).

The findings showed that the elderly subjects (male + female) in urban areas in the scales of mental quality of sleep (*p* < 0.001), sleep latency (*p* < 0.001), sleep duration (*p* = 0.024), daytime dysfunction due to sleepiness (*p* = 0.001) scored lower than elderly subjects (male + female) in rural areas, and as a result, they are in a better condition. It was found that there is a significant difference in overall sleep quality score between elderly subjects (male + female) in urban areas rural areas (*p* < 0.001). The elderly subjects (male + female) in urban areas in the scales of overall sleep quality scored lower than elderly subjects (male + female) in rural areas, and as a result, they are in a better condition (Table 3).

**Table 3:** Comparing the sleep quality of the elderly in urban and rural areas^a^

Variable	Subject	Urban	Rural	*p* value
Mental Quality Of Sleep	Men	0.663±0.645	1.617±0.630	< 0.001*
	Women	1.527±0.789	1.336±0.733	0.044*
	Total	1.145±0.845	1.467±0.699	< 0.001*
Sleep Latency	Men	0.762±0.883	1.211±1.212	0.002*
	Women	1.102±0.977	1.478±1.134	0.005*
	Total	0.950±0.950	1.352±1.176	< 0.001*
Sleep Duration	Men	1.144±0.798	1.173±0.614	0.760
	Women	1.074±0.469	1.282±0.538	0.001*
	Total	1.105±0.636	1.230±0.576	0.024*
Sleep Efficiency	Men	0.593±0.818	1.298±0.868	< 0.001*
	Women	1.455±0.869	1.051±0.981	< 0.001*
	Total	1.071±0.948	1.167±0.936	0.265
Sleep Disturbance	Men	0.661±0.542	0.661±0.66	0.030*
	Women	1.00±0.370	0.974±0.532	0.659
	Total	0.849±0.484	0.905±0.560	0.245
Sleep Medication Use	Men	1.245±0.691	0.971±0.645	0.002*
	Women	1.081±0.726	1.299±0.545	0.006*
	Total	1.154±0.714	1.144±0.615	0.869
Daytime Dysfunction Due To Sleepiness	Men	0.686±0.758	1.048±1.036	0.004*
	Women	1.095±0.77046	1.299±0.883	0.051
	Total	0.913±0.790	1.181±0.964	0.001*
Overall Sleep Quality	Men	5.754±1.753	8.134±3.353	< 0.001*
	Women	8.353±2.999	8.726±2.718	0.292
	Total	7.196±2.830	8.448±3.041	< 0.001*

Data are presented as the mean ± standard error of the mean

*P-value ≤ 0.05 considered significant

^a^Independent t-test was used

## Discussion

In the present study, we investigated the sleep quality of active and inactive elderly in two urban and rural environments of Bushehr. The study of sleep quality is an important clinical construct because it affects the daily functioning of people who complain of poor sleep quality. In addition, poor sleep quality can be an important symptom of many sleep disorders and medical disorders [30]. The findings of the present study showed that the sleep quality of active elderly in both male and female groups in both urban and rural areas was higher compared to the inactive group in these areas.

In this regard, the results of the study by Yang and Wang Stead (2014) showed that the time of awakening after the onset of sleep, the number of awakenings and the total number of activities after a session of moderate intensity aerobic training compared to people without exercise significantly decreased. As a result, a session of brisk walking with moderate intensity for approximately one hour improves sleep quality in elderly women [31]. In addition, exercise-induced weight loss improved sleep quality in obese older women with sleep disorders [1]. On the other hand, a study suggested that exercise can improve sleep latency, but no changes were observed for other sleep indicators, such as sleep duration, sleep efficiency, sleep disturbance, and daily functioning [32]. Also, Holfeld and Ruthig (2014) investigated the relationship between sleep quality and physical activity in the elderly. The findings of their study showed that sleep quality can cause higher levels of frequent physical activity over time, but physical activity does not cause better sleep quality. Therefore, sleep quality is important as an important factor in active lifestyle among the elderly [33]. According to the results of another study, it was shown that participating in sports programs creates a significant difference between different scales of sleep quality in postmenopausal women, which is effective in improving their sleep quality [34].

According to the findings of this study, it was found that active urban elderly men scored lower than inactive urban elderly men only in the sleep efficiency scale. Research findings show that despite the pressures of life in the rural environment due to environmental restrictions and lack of facilities, the need to perform physical activities to meet their needs along with the presence of beautiful nature, open space and clean air, their health is higher [35] and his issue can have a direct impact on the quality of sleep of people living in these environments. Zar et al. (2017) conducted a study with the aim of investigating and comparing the sleep quality of elderly men in Shiraz. The results of their study showed that in addition to the overall quality of sleep, there is a significant difference in other scales such as sleep efficiency and effectiveness, sleep disorders, the amount of sleeping pills consumed, and morning dysfunction among active and inactive elderly people, and the condition of active elderly people is favorable. It was more inactive than the elderly [36].

On the other hand, Myllymaki et al. (2012) stated that long exercise sessions may interfere with cardiac autonomic modulation in healthy men with moderate physical activity, and as a result, if the subjects are not used to this type of physical activity, the possibility of sleep disturbance is higher [37]. A piece of research by Khorzoghi and Sajjadian (2022) was conducted with the aim of investigating the effectiveness of pre-sleep exercises on sleep quality parameters and non-specific chronic back pain after sleep in elderly men. The findings indicated that exercising before going to sleep can be effective in improving sleep quality and non-specific chronic back pain [38]. Another study suggested that a simple and gentle exercise program can improve sleep quality for older adults [39]. Sleep is an essential part of life and a dynamic brain process that plays an important role in restoring physical and mental function. Normal sleep patterns vary significantly throughout life, with sleep disturbance and sleep problems appearing to increase in adulthood and with age.

Regular physical exercise may induce relaxation and increase core body temperature, which is useful for initiating and maintaining sleep [7, 40]. In the following, the findings of this study showed that the total number of active urban elderly men and women have better sleep quality than the total number of inactive urban elderly men and women, which is in line with the results of Bankar et al.‘s (2013) study regarding the effect of yoga exercise on improving the sleep quality of the elderly [41]. The findings of this study regarding the rural environment also showed that men, women and the total of active rural men and women have better sleep quality than the inactive group in the mentioned rural population. In this regard, Yuan et al. (2020) investigated the role of mental health and physical activity in the relationship between sleep quality and quality of life among rural elderly in China. The findings showed that considering that poorer mental health is somewhat related to worse quality of life among rural elderly, however, physical activity moderates its effect and improves sleep quality and mental health problems [42].

Also, in a population-based study by Bruto et al. (2020), it was shown that there is a relationship between adequate levels of physical activity and good sleep quality in middle-aged and elderly people living in rural areas [43]. In addition, it has been reported that there is a significant relationship between regular physical activity and reduction of insomnia symptoms in rural communities [44]. The results of a study comparing a city and a neighboring African village showed that rural residents slept on woven mats on the floor, had higher levels of physical activity during the day, and slept more people per room. Therefore, the sleep quality calculated in the rural environment was significantly weaker than the urban one. In addition, the pattern of exposure to light and coordination with it was reported to be different between the two communities, so that people living in rural areas coordinated their activities more with daylight hours, but residents of urban areas due to having more facilities due to development and progress., they were awake more hours of the night and were engaged in recreational or work matters, which can cause disturbances in the sleep of residents of urban areas, although the definite effect of this issue has not yet been proven and some aspects of industrialization show a higher quality of sleep in the urban population. have given [45]. It has been reported that intense exercise does not have a positive effect on various aspects of sleep quality [46]. As a result, vigorous physical activity before bedtime may negatively affect sleep. Also, in a study conducted on elderly with mild sleep disorder, it was reported that 8 weeks of water exercise was effective only on some sleep parameters, including less time for sleep onset delay and better sleep efficiency [47].

While according to the results obtained from the present study, men, women and the total of active men and women have better sleep quality than the inactive elderly in the whole urban and rural scale, which is in agreement with the results of some studies in the field of sports such as walking and its positive association with the quality of sleep is the same [48–50]. This study has generally examined the effect of physical activity on sleep quality and no specific type of exercise has been mentioned. In the current study, the activeness of the samples was determined based on their self-report, and the intensity of the activity and their awareness of the impact of physical activity on health were not evaluated, which is one of the limitations of the present manuscript. However, since both of these cases can affect the level of mental and physical health and, as a result, probably the quality of their sleep, it can be investigated in future research. Considering that this study was conducted on the elderly, the results cannot be generalized to other age groups. Also, in this study, the time of physical activity during the day and night is not mentioned. In addition, subjects were not influenced by laboratory conditions to obtain more accurate results.

Considering the importance of sleep quality in this group of people in the society, it is suggested that screening and early and timely diagnosis of sleep problems in the elderly population in the country should be implemented and suitable intervention programs should be designed. In addition, the findings of this study can be important in order to pay attention to the importance of physical activity and its effect on the quality of sleep in the elderly. Also, due to the lack of studies in the field of examining the level of physical activity with the quality of sleep of the elderly in urban and rural areas, the findings of this research have examined the importance of this issue. Whether different levels of physical activity can make a difference in sleep quality needs further investigation. However, one limitation of our study was that active people were classified as active people based on doing physical activity 3 days a week and their sleep quality was investigated, but the type of exercise and its effect on sleep quality was not investigated.

The environment is one of the effective factors in the state of sleep quality and it seems that the facilities of the living environment of elderly people can be the basis for more participation in physical activity and because of improving the quality of sleep of this age group. On the other hand, considering that it is easier to control the environmental factors, it seems that the problems related to sleep caused by the physical environment can be solved to a great extent [47]. Therefore, it is necessary for managers related to health in different urban and rural areas to take effective measures to improve their health by removing environmental barriers related to the elderly.

## Conclusion

Overall, the results of this research showed that in both groups of active elderly men and women, sleep quality was higher than that of inactive people, and this issue was true in both urban and rural residential areas. However, according to the results of the current research, active people had better sleep quality in both rural and urban areas, and it seems that physical activity is an essential factor in having a healthy lifestyle, which can have a positive effect on the health components of the elderly, including the quality of their sleep, so it can be said that exercise and physical activity can have a positive effect on people’s sleep quality regardless of location. As in our study, the obtained results confirm that, in general, the total number of active male and female seniors in the overall urban and rural scale had better sleep quality than the total number of inactive male and female seniors in the overall urban and rural scale He did not understand that this issue can be addressed in the future researches.

## Data Availability

The data that support the findings of this study are available from the corresponding author, [author initials], upon reasonable request.
